# Descending Perineum Associated With Pelvic Organ Prolapse Treated by Sacral Colpoperineopexy and Retrorectal Mesh Fixation: Preliminary Results

**DOI:** 10.3389/fsurg.2018.00050

**Published:** 2018-09-20

**Authors:** Aude Nessi, Aminata Kane, Etienne Vincens, Delphine Salet-Lizée, Karine Lepigeon, Richard Villet

**Affiliations:** ^1^Département Femme Mère Enfant, Centre Hospitalier Universitaire Vaudois (CHUV), Lausanne, Switzerland; ^2^Visceral and Gynaecologic Surgery Unit, Diaconesses Hospital, Paris, France

**Keywords:** descending perineum syndrome, Sacral colpoperineopexy, obstructed defecation syndrome, quality of life, mini invasive surgery

## Abstract

**Introduction and hypothesis:** Descending Perineum Syndrome (DPS) is a coloproctologic disease and the best treatment for it is yet to be defined. DPS is frequently associated with pelvic organ prolapse (POP) and it is reasonable to postulate, that treatment of POP will also have an impact on DPS. We aimed to evaluate the subjective satisfaction and improvement of DPS for patients who have undergone a sacral colpoperineopexy associated with retrorectal mesh for concomitant POP.

**Methods:** This retrospective cohort study, conducted between February 2010 and May 2016 included all women who had undergone surgery to treat POP and DPS. Improvement of POP was assessed clinically and subjective satisfaction was assessed with a survey.

**Results:** Among the 37 operated patients, 31 responded to the questionnaire and 77.4% were satisfied with this surgical procedure. 94.6% were objectively cured for POP. There was a 60% improvement rate for constipation, 63.5 and 68% were cured or improved for ODS and the need for digital maneuvers respectively.

**Conclusion:** Sacral colpoperineopexy associated with retrorectal dorsal mesh appears to objectively and subjectively improve POP associated with DPS.

## Summary

This retrospective cohort study evaluates objective and subjective improvement in patients with Pelvic organ prolapse associated with descending perineum syndrome treated by an original surgery.

## Introduction

Perineal descent PD which was first described by Parks et al. (1) in 1966, is defined as descent of the anal margin under the line passing through the ischial tuberosity on clinical examination (1–3) and is characterized by swelling of the perineum. Only 14% of patients with PD will be symptomatic with coloproctologic symptoms such as constipation, obstructive disease syndrome (ODS) and incomplete bowel emptying which frequently requires digital maneuvers (2, 3). Such a combination is defined as Descending Perineum Syndrome (DPS) which is associated with a history of chronic straining and a sensation of incomplete defecation which is sometimes followed by a feeling of obstruction. There can be complaints of anal bleeding, loss of mucus, perineal pruritus or pain. Sometimes digital maneuvers are required to pass stool (1, 3, 4) and severe cases are often associated with anal incontinence (1, 4, 5).

DPS can deteriorate over the years, progressing from isolated ODS to fecal incontinence which can lead to a vicious cycle: the more straining, the more stretching of the levator ani muscle with aggravation of ODS. Several potential causes for DPS have been described: straining, neuropathic degeneration of muscle due to aging and trauma of the pelvic floor muscles and the pudendal nerves during labor and pregnancy. The patient's prolonged efforts to excrete will worsen the perineal descent. The anterior rectal wall mucosa bulges into the anal canal, which mimics obstruction at this point. Some bleeding may appear as well as loss of mucus, then further straining follows. Finally the anterior rectal wall mucosa protrudes as well.

Pudendal neuropathy may also be the consequence of PD. DPS at its last stage is characterized by rectal prolapse and severe denervation (6) of the pudendal nerve consequent to up to 20% (5) overstretching with subsequent fecal incontinence as well as neuropathy (7).

PD can be measured with a perineocaliper® although this device tends to underestimate the descent, particularly in obese patients (8, 9).

Radiologically, PD is defined as the descent of the anorectal junction below the pubococcygeal line extending from the inferior border of the pubic symphisis to the tip of the tailbone during straining (10). Initially, colpocystogram and defecography were used for imaging of PD, but recently, dynamic MRI has been demonstrated to be more accurate with less exposure to ionizing radiation (11).

First line therapy consists of dietary recommendations and use of laxatives (hydrophilic laxative or irritant suppository) to facilitate the rectal emptying (1, 4). The second line would be biofeedback and pelviperineal physiotherapy, which aims to reinforce the muscle and prevents further damage. However, there is a low response rate to these conservative approaches and only patients in an early stage of DPS seem to benefit from it (12). For the surgical option, different procedures have been suggested (13–16) but no consensus has been reached so far, whether a perineal, transanal, transvaginal, or abdominal approach would be preferable.

The aim of this study was to evaluate satisfaction and improvement of DPS patients with concomitant POP who have undergone a sacral colpoperineopexy associated with a tension free retrorectal mesh.

## Methods

### Study population

The Institutional Review Board of the Diaconesses Croix Saint Simon Hospital of Paris approved this study protocol. We included all women treated between February 2010 and May 2016 for POP and DPS by sacrocolpoperineopexy with placement of 3 or 4 prosthetic polypropylene meshes (inter vesicovaginal, ventral rectopexy, dorsal rectopexy, posterior mesh with uterosacral fixation). Patients were selected based on data available from hospital files.

### Perioperative evaluation

We used a prospective record file, specifically designed for pelviperineology patients, in order to document history and clinical examination of the urological, gynecological and coloproctologic compartment. All perineum-descending symptoms were recorded and POP was evaluated with the POP-Q system test (17) during Valsalva's maneuver. PD was clinically suspected in the presence of elongation of the ano vulvar distance during straining and thinning of the intergluteal fold, later confirmed either by MRI and/or defecography. Patients with cystocele, urinary stress incontinence or occult stress urinary incontinence were referred for urodynamic assessment. All patients with fecal incontinence underwent endoanal ultrasound and Ano-Rectal Manometry (ARM).

Patients with POP and associated DPS were offered a standardized laparoscopy as described below.

All patients had postoperative assessment by the surgeon at 5 weeks and all patients received the questionnaire evaluating satisfaction by mail.

### Surgical procedure

**Figure 1:**
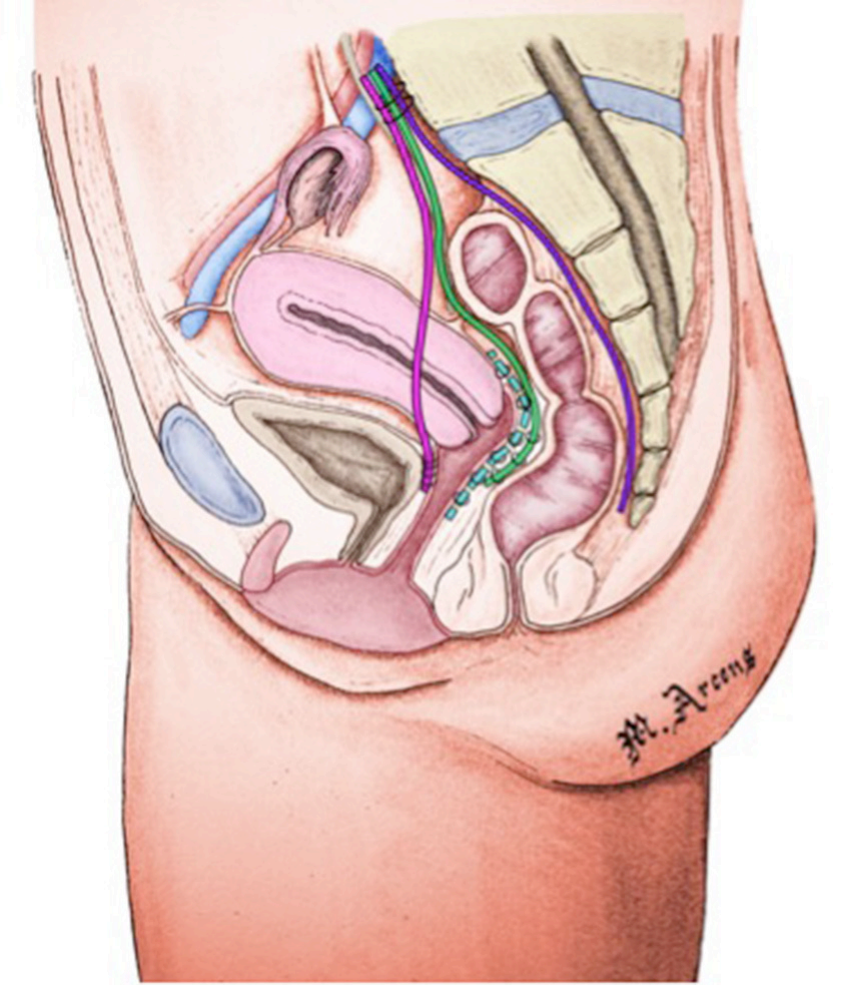
Surgical procedure. Image copyright: Marc Arcens, used with permission.

Our standardized technique for POP/DPS (Figure 1) repair consists of:

- Placement of an anterior mesh between bladder and vagina for bladder suspension,
- Placement of a dorsal mesh between rectum and sacral concavity to fix the perineum,
- Placement of a posterior mesh with uterosacral fixation in case of rectocele
- Placement of a ventral tension free mesh between vagina and rectum fixed to the promontory, in case of exteriorized rectal prolapse or stage IV intra rectal intussusception.

The patient is in supine position; legs spread apart and slightly flexed. Traditional laparoscopic procedure or open technique consists in insertion of a 10 mm umbilical trocar, 5 mm iliac trocar in both sides, and 5/10 mm halfway between the umbilic and pubic symphysis. We first expose the promontory and insert a polyester suture in the prevertebral ligament. After identifying the right ureter, we proceed to peritoneal incision in the right border of the mesorectum, retrorectal dissection behind the rectal fascia and then the dissection is continued toward the *levator ani* distally and toward the tailbone laterally and medially. A mesh is fixed laterally, with non- absorbable braided sutures to the levator muscle. The mesh was fashioned to fit perfectly the presacral concavity and fixed to the promontory tension-free. The Douglas pouch is then incised and the rectovaginal space is dissected until reaching the central fibrous perineal body. The ventral prerectal mesh is fixed laterally to the levator muscles, to the anterior rectal and to the promontory without tension. We perform the vesico vaginal dissection, suture the vesico vaginal mesh to the vagina and tie it up to the promontory with tension. At the end, we perform a reperitonisation (15) (Figure 1).

All the procedure were performed by laparoscopy or Da Vinci robotic assisted laparoscopy; there were no case of conversion to laparotomy or with perioperative complication.

### Postoperative objective assessment

Each patient had postoperative examination by their surgeon between 5 and 12 weeks. Pain, coloproctologic, urinary symptoms were evaluated. The patient was examined with the search of mesh complication, evaluation of results with a POP-q testing, quality of the scar, and anal digital examination.

### Subjective evaluation

In February 2016, we mailed a questionnaire (Appendix) to all eligible patients with a pre stamped envelope and a letter informing them that they would get a call in the event that we would have no response. In April 2016 we mailed the same questionnaire a second time and in May we called the non-responders.

### Statistical analysis

Differences in categorical variables between the groups were analyzed using chi-squared test (or Fisher's exact test when appropriate). Quantitative variables were reported as mean and deviation standards (or median and interquartile ranges when appropriate). Normal quantitative variables were compared using student *t*-test. Analyses were performed using STATA version 14 (Copyright 1985–2015 StataCorp LP). Statistical significance was defined as *p* < 0.05.

## Results

### Preoperative sample characteristics

Based on our hospital files, between February 2010 and May 2016, 37 patients had abdominal sacrocolpoperineopexy associated with positioning of a retrorectal mesh. Mean age of evaluated patients was 53.9 years (40–84), mean parity was 2.6 (1–5), and 52% had 3 children or more, 6 patients (16.2%) had instrumental deliveries. 18/37 patients (48.6%) were postmenopausal and one was on hormone replacement treatment. Mean BMI was 23.9 (17.6–31.2), 12/37 patients (32%) had a history of surgery for prolapse and 6/37 patients (16.2%) a history of ano-rectal surgery.

Preoperative bowel symptoms were present in all patients (100%), this included constipation in 33 patients (89%), ODS in 26 patients (70%), gas incontinence in 14 patients (38%), 16 (43%) had incomplete excretion and 29 patients (78.4%) required digital maneuvers to properly empty their bowel. Thirteen patients (35%) presented with clinical stress urinary incontinence (Table 1).

**Table 1:** Patients characteristics.

**Characteristic**	**Value (range or %)**
Age (year)	53.9 (40–84)
No of vaginal deliveries	2.6 (1–5)
Post-menopausal	18 (50%)
History of surgery for prolapse	12 (32%)
History of anorectal surgery	6 (16%)
Active sexual life	20 (55%)
**SYMPTOMS**	
Constipation	33 (89%)
Fecal incontinence	14 (38%)
ODS	26 (70%)
Digital maneuver	29 (78%)
Incomplete exoneration	16 (43%)
Sensation of incomplete evacuation	17 (46%)
SUI	13 (35%)
**PHYSICAL EXAMINATION**	
BMI	23.9 (17.6–31.2)
Prolapse stage I	5 (13.2%)
Prolapse stage II	16 (43.6%)
Prolapse stage III	16 (43.6%)
Occult SUI	7 (19%)

All patients had pelvic organ (prolapse POP) according to the International Urogynaecological Association with stage 1 in 5 patients (13.6%), stage 2 in 16 patients (43.2%), and stage 3 in 16 patients (43.2%). Eighteen patients (48.6%) had stage 2 or 3 rectocele and, 22/37 (59.5%) presented with stage 4 intra-rectal intussusception (IRI) and 6 had (16.2%) rectal prolapse.

Twenty-five patients (67.6%) had a colpo MRI, 15 (40.5%) a defecography, 16 (43.2%), an ARM, and 24 (65%) patients had urodynamic assessment. In 30 patients (81%) we performed ventral rectopexy in addition to the main surgery.

### Results of surgery

We had 4 postoperative complications: urinary retention resolve few days after surgery with bladder catheterization, an evisceration at the level of left trocar, exposure keratopathy and a third degree umbilical burn occurred with the water.

We detected a 94.6% cure rate with a stage 0 or 1 POP at postoperative physical examination. One patient had a residual stage 2 cystocele with a good correction of hysteroptosis and another patient had a low rectocele for which she underwent vaginal perineal repair 1 year later. Rectal prolapse was cured in 100% of cases. No case of mesh rejection was observed in this small sample.

### Questionnaire-based satisfaction assessment

In February 2016, we received 21/37 responses (56.8%). In April 2016, we mailed the same questionnaire a second time and received 3 back (8.1%). We successfully called 7 remaining patients (18.9%) in May 2016. Six (16.2%) patients who had changed their address were lost to follow-up. Overall mean follow up was 38 months (11–80).

Concerning overall satisfaction of the 31 responders, 24 patients (77.4%) were satisfied with the subjective outcome of the procedure.

We investigated if medical history had an influence on the satisfaction and did not find any statistically significant association between satisfaction and menopause, history of anorectal and POP surgery, fixed descending perineum, ventral mesh rectopexy, and hysterectomy. Patients at 3-year follow up are less satisfied 62, 5% than at 1 year follow up 100% (Table 2).

**Table 2:** Satisfaction despite year of follow up.

**Length of follow up (year)**	**Satisfied (*n*)**	**%**
1	2/2	100
2	10/14	71.5%
3	5/8	62.5%
4	4/4	100
5	1/1	100
6	2/2	100

Twenty-seven patients with constipation responded to the questionnaire; one was cured (4%), 15 patients (56%) were improved, nine (33%) did not note any change and two (7%) had worsening of their symptoms. Twenty-two patients with pre-operative ODS responded to the questionnaire; Two (9%) were cured, 12 (54.5%) improved, seven (32%) did not note any change and one (4.5%) had deteriorated. Twenty-two patients requiring digital maneuvers responded to the questionnaire, seven (32%) were cured, eight (36%) improved and seven (32%) did not note any change.

## Discussion

Descending perineum is an anatomic description (1), and is not only frequently associated with anorectal symptoms, therefore defining the DPS, but also with rectocele which is known to induce straining (18). In our study population, all patients with DPS had associated POP. We hypothesized that restoration of the anatomy with the correction of both DP and POP may improve not only POP related symptoms, but also DPS, which could break the vicious circle at its root.

Nevertheless, this hypothesis does not apply to all patients and following the “*primum movens”* principle, we would suggest using a personalized strategy. This would also explain the variability of results concerning surgery for DPS in the literature.

However, we did not know if this surgical procedure was adapted for every kind of DPS. Most studies argue that DPS must be treated by conservative measures, such as hygieno-dietetic recommendations, pelvic floor exercises (PFE) and biofeedback (1, 12, 18). Harewood et al. (12) showed that efficacy of biofeedback seems to depend on the extent of perineal descent (12); patients who responded to PFE and biofeedback had a smaller perineal descent (mean 3.3 cm) than non-responders (mean 4.9 cm). Numerous studies demonstrated that the worse the perineal descent, the more difficult is efficacious treatment (12). They also found associated features between DPS and the female gender (96%), multiparity with vaginal delivery (55%) and with hysterectomy or vaginal cystocele and/or rectocele repair (74%). Chang et al. (19) found a statistically significant association between DPS, age and increasing number of vaginal deliveries, whereas surgical history did not have any impact.

In our study, we observed a trend toward a negative impact of previous surgical history. Indeed, even if it does not reach statistical significance, it appears that 87% of the satisfied women have no history of POP surgery compared to only 50% with POP surgery history (*p* = 0.05).

In our study group, 94.6% of the patients who suffered from POP were objectively cured, which is consistent with findings in the literature (20). Cundiff et al. (16) reported 19 patients cured by abdominal sacralcolpoperineopexy performed for PD with concomitant vaginal vault prolapse and they concluded that this procedure is reliable. Sacralcolpopexy is considered the gold standard to treat POP in young women who are sexually active. Bacle et al. (20) showed a recurrence rate of 11.5% in a cohort of 501 patients with a 10-year follow-up. In a review of 11 laparoscopic studies that included 1197 patients, Ganatra et al. (21) reported a 10% recurrence rate for POP, which is similar to the results in our study.

Several studies assessed the impact of ventral mesh rectopexy for complex rectocele associated with constipation. Wong et al. (22) described 84 women who underwent laparoscopic ventral mesh rectopexy for symptomatic complex rectocele. Eighty-eight percent reported an improvement and there was a significant decrease of ODS (83–46%). Formijne Jonkers et al. (23) studied functional results on 245 women who underwent laparoscopic ventral rectopexy, and found a significant reduction of 34% in defecation symptoms. In our study, we found an even higher reduction of 51% in defecation symptoms.

To our knowledge, no study has yet evaluated the impact of sacrocolpoperineopexy on PD. Recently, Renzi et al. (14) described a new procedure of transverse perineal support. They treated 12 patients and obtained a significantly lower PD at the postoperative X-ray and MRI defecography for fixed and dynamic PD (*p* = 0.02 and *p* = 0.0004 respectively). In fact Renzi et al. restored only the perineal support and they found no change in rectocele and/or rectal intussusceptions. They concluded that it is conceivable to associate their technique with an additional intervention treating rectocele or rectal prolapse. Our procedure intends to follow that recommendation and to additionally restore the perineal prolapse anatomy overall. However, DPS caused by pudendal neuropathy may not improve sufficiently with surgery. Interestingly, Snook et al. (24) have studied Pudendal Nerve Terminal Motor Latency (PNTML) in 71 women who had a vaginal delivery; They showed an increase of PNTML in 42% of women 48–72 h after labor with a normalization after 2 months in 60% of cases. Studies including PNTML testing before and after surgery would be required to support this hypothesis.

The strength of our study is that, to our knowledge, it is the first to investigate anatomic outcome and satisfaction of surgical treatment of DPS associated with POP. Limitations are the retrospective design and the small sample size. However, to our knowledge, this study is the largest available so far.

*Primum movens* is sometimes difficult to be determined and probably a more personalized approach to patients with DPS, focusing on its cause, would improve the results and patients satisfaction. Maybe early surgical management of PD before occurrence of DPS would be adequate.

## Conclusions

Sacral colpoperineopexy associated with the dorsal mesh appears to be an effective procedure in the treatment of pelvic organ prolapse associated with descending perineum in regards of DPS symptoms patient satisfaction.

A prospective long term study on the outcome of patients with DPS who have undergone this kind of surgery would be needed to confirm our results.

## Author contributions

AN: project development, data collection and analysis, manuscript writing and editing; AK and RV: project development, manuscript editing; EV and DS-L: manuscript editing; KL: management data analysis, manuscript editing.

### Conflict of interest statement

The authors declare that the research was conducted in the absence of any commercial or financial relationships that could be construed as a potential conflict of interest. The handling Editor declared a shared affiliation, though no other collaboration, with the authors AN and KL.

## Appendix

### Questionnaire

1. Are you satisfied by the intervention?

□ yes □ no

2. Would you reiterate the operation?

□ yes □ no

3. Would you advise this intervention to a relative?

□ yes □ no

4. Is there an improvement of symptoms of constipation?

□ cured □ improved □ no change

5. Is there an improvement of ODS?

□ cured □ improved □ no change

6. Do you need to perform digital removal of faeces when defecating?

□ cured □ improved □ no change

7. Do you use laxatives?

□ yes □ no

If yes at what frequency:
